# Melatonin Protects the Apoptosis of Sheep Granulosa Cells by Suppressing Oxidative Stress via MAP3K8 and FOS Pathway

**DOI:** 10.3390/genes14051067

**Published:** 2023-05-11

**Authors:** Bo Zhai, Xu Li, Zhongli Zhao, Yang Cao, Xinxin Liu, Zheng Liu, Huihai Ma, Wenfa Lu

**Affiliations:** 1 Institute of Animal Science, Jilin Academy of Agricultural Science, Changchun 136100, China4513046@163.com (X.L.);; 2 College of Animal Science and Technology, Jilin Agricultural University, Changchun 130118, China

**Keywords:** melatonin, apoptosis, sheep, granulosa cells, oxidative stress

## Abstract

Melatonin is not only a highly effective active oxygen scavenger but also an important reproductive hormone. Melatonin has a regulatory effect on animal reproduction, especially on the ovaries. It can affect the proliferation and apoptosis of cells in follicles. However, the mechanisms of the dual antioxidation and anti-apoptosis effects of melatonin on granulosa cells are still not clear, especially in sheep. Therefore, we investigated the mechanisms of the protective effect of melatonin against oxidative damage in granulosa cells. At a concentration of 250 µmol/L, H_2_O_2_ promoted granulosa cell apoptosis; however, 10 ng/mL melatonin effectively alleviated the pro-apoptotic effect of H_2_O_2_. Furthermore, through the application of high-throughput sequencing technology, we identified 109 significantly differentially expressed genes (35 upregulated and 74 downregulated genes) involved in the protective effect of melatonin against apoptosis. The expression levels of nine related genes, i.e., *ATF3*, *FIBIN*, *FOS*, *HSPA6*, *MAP3K8*, *FOSB*, *PET117*, *DLX2*, and *TRIB1*, changed significantly. *MAP3K8* and *FOS* gene overexpression impacted the protective effect of melatonin in granulosa cells; the two genes exhibited an upstream and downstream regulatory relationship. Our findings indicated that melatonin alleviated H_2_O_2_-induced apoptosis in sheep granulosa cells through the MAP3K8-FOS pathway.

## 1. Introduction

Melatonin (MT) is a neuroendocrine hormone initially discovered in the bovine pineal gland. It is not only a highly effective reactive oxygen species (ROS) scavenger but also an important reproductive hormone. As a natural fat-soluble antioxidative factor, melatonin scavenges different types of free radicals, such as hydroxyl radicals (-OH) and hydrogen peroxide (H_2_O_2_) [1]. Melatonin can maintain mitochondrial function by removing or reducing oxidative stress in mitochondria, thereby reducing the occurrence of subsequent apoptosis. The effects of melatonin on the reproductive performance of female animals mainly manifest in sexual maturation, hormone secretion, follicular development, luteal function, ovulation, pregnancy, and childbirth [2]. In addition to its synthesis in the pineal gland, melatonin is also synthesized in the cumulus oocyte complex and granulosa cells and participates in follicular development [3]. The high concentration of melatonin in follicular fluid is very important for inhibiting follicular atresia. Melatonin can protect oocytes from free radical damage during ovulation, allowing the full development of preovulatory follicles.

Granulosa cell apoptosis is a key influencing factor for follicular atresia [4]. Melatonin exerts anti-apoptotic effects in the process of follicular atresia in a variety of animals, inhibits granulosa cell apoptosis, and promotes follicular development [5,6,7,8,9,10,11]. Melatonin can not only directly affect the growth status of granulosa cells and the production of steroid hormones but also indirectly alleviate the damage caused by extracellular stimuli [11,12,13,14]. Melatonin can affect the proliferation and apoptosis of bovine granulosa cells and stimulate the secretion of progesterone through melatonin 2 (MT2) and melatonin 1 (MT1) receptors in vitro [15]. It can also regulate the proliferation and apoptosis of chicken granulosa cells through mTOR signaling pathway activation [16]. Melatonin has a significant reparative effect on the exogenous damage to granulosa cells. When granulosa cells are treated with bisphenol A [17] and BMP6 (Recombinant Human Bone Morphogenetic Protein 6) [18], melatonin effectively resolves the induced granulosa cell proliferation and steroid hormones production. Melatonin affects the proliferation and apoptosis of sheep granulosa cells under heat stress [19].

Studies on the regulation of granulosa cells by melatonin mainly focus on swine, cattle, rats and other animals. However, the protective effect of melatonin from oxidative stress in sheep granulosa cells has not been reported. This study on the ability of melatonin to alleviate H_2_O_2_-induced apoptosis in sheep granulosa cells through the MAP3K8-FOS pathway provides a theoretical reference for the role of melatonin in the regulation of sheep reproductive functions.

## 2. Materials and Methods

### 2.1. Ethics Statement

The collection of test samples in this research was approved by the Animal Care and Use Committee of Jilin Agricultural University.

### 2.2. Isolation and Culture of Granulosa Cells

Follicles (3–5 mm) isolated from the ovary of small tailed han sheep (six years old) were soaked in 75% alcohol and placed in precooled Dulbecco’s phosphate-buffered saline (DPBS). The follicles were cut open in petri dishes (containing DPBS), and the inner wall of the follicle was gently scraped to obtain parietal granulosa cells, which were collected in a 15-mL centrifuge tube. After centrifugation at 1000× *g* r/min for 5 min, the supernatant was discarded, and the pellet was resuspended in a complete medium (DMEM/F12), centrifuged and washed twice. A cell suspension in the complete medium was spread on a culture dish, and the dishes were incubated in a cell incubator (37 °C, 5% CO_2_) for subsequent experiments.

### 2.3. Cell Viability Assay

The cells were seeded in 96-well plates (3 replicates for each group) at a seeding density of 1 × 10^4^ cells/well. After reaching confluency and attaching to the wells, the cells were cultured in a serum-free medium with different concentrations of H_2_O_2_ (0, 100, 200, 250, 300, 400, 500 umol/L) and melatonin (0.1, 1, 10, 100, 1000 ng/L), and MTT (3-(4,5-Dimethylthiazol-2-yl)-2,5-diphenyltetrazolium bromide), diluted with 5% PBS was added to the wells at 20 μL/well and removed after 4 h. After an appropriate amount of DMSO (Dimethyl sulfoxide) was added, cell viability was detected at 490 nm.

### 2.4. Flow Cytometric Analysis of Apoptotic cells

The granulosa cells from the groups with the control, H_2_O_2_ treatment and H_2_O_2_+Melatonin treatment, were transferred to 1.5 mL centrifuge tubes, and 200 μL of 1× Binding Buffer was added to each tube in accordance with the BD Apoptosis Detection Kit. Propidium iodide (PI) was added to one tube of cells, and fluorescein isothiocyanate (FITC) was added to another tube of cells. Five microliters each of FITC and PI were added to the cells in each tube of different treatments and mixed by inversion, and the cells were incubated in a cell culture incubator for 20 min. Then, 200 μL of 1× Binding Buffer was added to each tube, and the cells were filtered through a nylon mesh sieve. The percentage of apoptotic cells was determined by flow cytometry.

### 2.5. Real Time Quantitative Polymerase Chain Reaction (qRT-PCR) Analysis

The total RNA was isolated from the cells, using the TRIzol™ reagent (Invitrogen, Waltham, MA, USA), and 1 μg was employed to generate cDNA, using a PrimeScript RT reagent Kit (Takara, Kusatsu, Japan). Next, qRT-PCR was performed using SYBR^®^ Premix Ex Taq™ II (Takara, Kusatsu, Japan) on a Real-Time PCR Detection System (Santa, TX, USA). The primer sequences are shown in Table 1. The relative expression of genes was calculated, using the 2^−ΔΔCt^ method [20].

### 2.6. High-Throughput Sequencing Analysis

One milliliter of TRIzol (Invitrogen, USA) was added to sheep GCs with a different treatment for extracting the total RNA, and the quality was analyzed using an Agilent Bioanalyzer 2100. The total RNA extracted from the different treatment GCs was stored at −80 °C and used for the high-throughput sequencing. After the concentration was measured using a Qubit 2.0 Fluorometer, a library was constructed by Novogene Bioinformatics Technology Co., Ltd. (Beijing, China) and sequenced using the Illumina HiSeq 2500 platform.

The raw image file, which had now passed through base identification and error filtering, finally resulted in the original sequencing fragment that could be used for analysis, which we call Reads. Sequencing raw reads may contain unqualified reads such as low overall quality, sequencing primers, and low-end quality, which are likely to have a certain impact on the quality of analysis, so they must be filtered to obtain clean reads that can be used for data analysis, and Seqtk (https://github.com/lh3/seqtk accessed on 15 September 2020) should be used for filtering. To render the gene expression levels of different genes and different samples comparable, reads are converted into FPKM (Fragments Per Kilobase of exon model per Million mapped reads) for gene expression standardization. We first used Stringtie (version:1.3.0) to count the number of fragments of each gene after Hisat2 alignment, and then normalized the number of fragments using the TMM (trimmed mean of M values) method, and finally used a perl script to calculate the FPKM value of each gene. Subsequently, edgeR was adopted for genetic differences analysis between samples, and multiple hypothesis test correction was carried out after the *p*-value was obtained, and the threshold of the *p*-value was determined by controlling the FDR (False Discovery Rate), and the corrected *p*-value was q-value. At the same time, we calculated the difference expression multiple, i.e., fold change, based on the FPKM value.

Gene Ontology (GO) analysis was applied to explore the functions of differentially expressed genes identified in this study. GO analysis organizes genes into hierarchical categories and can uncover gene regulatory networks on the basis of biological processes and molecular functions (http://www.geneontology.org accessed on 30 March 2023). Specifically, the two-sided Fisher’s exact test was used to classify the GO category, and the GO annotation list was by chance greater than expected. An FDR was calculated to correct the *p*-value. We computed *p*-values for GOs enriched among differentially expressed genes (the recommended *p*-value cutoff is 0.05). Pathway analysis was employed to place differentially expressed genes according to the Kyoto Encyclopedia of Genes and Genomes (KEGG, http://www.genome.jp/kegg/ accessed on 30 March 2023). Fisher’s exact tests were also used to identify pathways, and the threshold of significance was defined by the *p*-value. The enrichment was calculated in a similar manner as the GO analysis. 

### 2.7. Western Blot Analysis

The total protein was extracted from sheep ovine granulosa cells using RIPA cell lysate. After protein quantification, 30 μg of total protein was subjected to SDS-PAGE gel electrophoresis, transferred to a membrane, and blocked. The membrane was incubated with diluted primary antibodies for Bcl2 (1:500), Bax (1:1000), Caspase-3 (1:1000), and β-actin (1:10,000) at 4 °C overnight. After incubation with the secondary antibody at room temperature for 1 h, ECL, the membrane was incubated in a chromogenic solution for 30 s, and the protein bands were visualized using a Chemi Scope imaging system (CLiNX Science Instruments, Shanghai, China). The grayscale values of the protein bands were analyzed using ImageJ, and the relative protein expression levels were calculated using β-actin as an internal reference.

### 2.8. Overexpression Vector Construction

Firstly, the candidate gene and recombinant expression vector were amplified and identified, and the PCR product was linked to the pcDNA3.1 (−) eukaryotic expression vector after digestion. The mRNA of the recombinant expression vector was tested.

### 2.9. Statistical Analysis

SPSS 22.0 (2019)software was used for qRT-PCR, Western blot, MTT and Flow cy-tometric results data analysis, and the Hisat (version:2.0.4) analysis was used for the sequence data. GraphPad Prism 7.0 (2020) software was used for graphing. The independent sample t test and the analysis of the variance were used to identify significant differences between groups (Control, Melation, Melation+H_2_O_2_). GraphPad Prism 7.0 software was used for graphing. Experimental data are expressed as “mean ± standard error”, and all experiments were divided into three groups with three samples for each group. A significant difference is indicated by *p* < 0.05.

## 3. Results

### 3.1. Melatonin Protects against H_2_O_2_-Induced Apoptosis in Sheep Granulosa Cells

**Figure 1:**
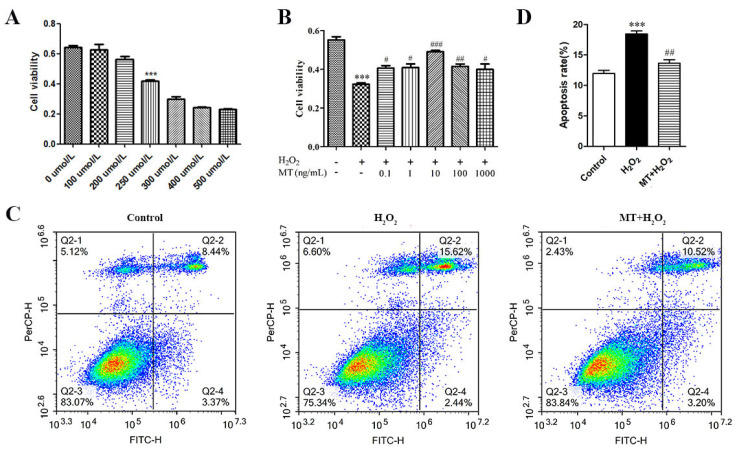
Melatonin represses H_2_O_2_ −induced apoptosis in cultured granulosa cells. (**A**) The effects of different concentrations (0, 100, 200, 250, 300, 400, 500 uM) of H_2_O_2_ on the viability of sheep granulosa cells. (**B**) The effects of different concentrations (0, 0.1, 1,10, 10, 100 and 1000 ng/mL) of melatonin on the viability of sheep granulosa cells with 250 uM H_2_O_2_ treatment. (**C**) Apoptosis levels measured by flow cytometry assay. (**D**) Apoptosis rate of flow cytometry assay. The results were acquired from three independent experiments and are presented as the mean ± SD. For *** *p* < 0.01 vs. the negative control group, the difference is extremely significant; for # *p* < 0.05 (##,### *p* < 0.01,) vs. the H_2_O_2_ group, the difference is significant or extremely significant.

To determine the concentration for exogenous treatment and detect the effects of treatment on granulosa cell proliferation and apoptosis, the MTT assay and double-staining flow cytometry were used. After 3 h of treatment with different concentrations (0–500 μM) of H_2_O_2_, the cell viability decreased with the increase in the concentration of H_2_O_2_ in a dose-dependent manner; the median lethal dose (LD50) was 250 μM. After treatment with the serum-free medium containing different concentrations of melatonin (1–1000 ng/mL) for 24 h, the cell viability in the 0.1 ng/mL and 1 ng/mL treatment groups increased significantly (*p* < 0.05), and cell viability in the 10 ng/mL treatment group demonstrated an extremely significant increase (*p* < 0.01). When concentrations were higher than 10 ng/mL, cell viability decreased (Figure 1).

The cells were cultured in a serum-free medium containing 10 ng/mL melatonin for 24 h and treated with 250 μmol/L H_2_O_2_ for 3 h.Double-staining flow cytometry indicated that the apoptotic rate for the group treated with melatonin was significantly reduced (*p* < 0.01), that the addition of melatonin effectively protected against sheep granulosa cell apoptosis after H_2_O_2_ treatment.

### 3.2. High-Throughput Sequencing Analysis

After we excluded the adaptors and N-containing and low-quality reads from raw reads obtained by high-throughput sequencing, a total of 57,693,778 clean reads were obtained for the blank group (N), accounting for 92.83%. A total of 64,057,360 clean reads were obtained for the H_2_O_2_ treatment group, accounting for 93.38%, and 61,994,733 clean reads were obtained for the melatonin+H_2_O_2_ (M+H) treatment group, accounting for 92.80%. The obtained clean reads were compared with reads in the Oar-v3.1 RefSeq database, and 27,055 annotated transcripts were obtained.

**Figure 2:**
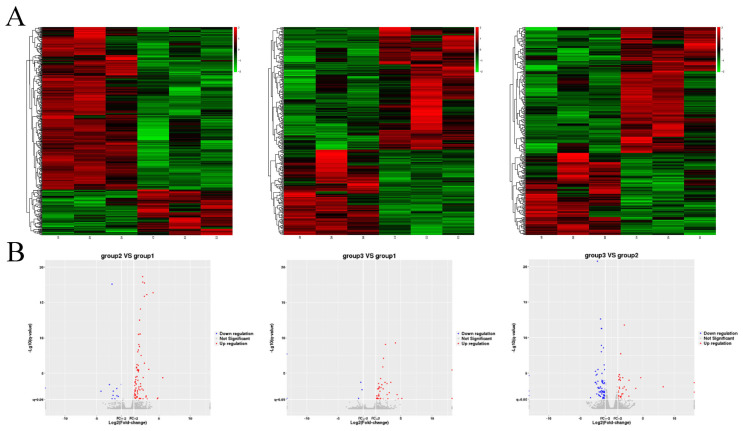
Hierarchical Clustering of Differentially Expressed Genes. (**A**) Differential gene heatmap. C and H represent the granulosa cells’ samples of control and H_2_O_2_ treatment, respectively. M represents the granulosa cells’ samples of H_2_O_2_+Melatonin treatment. The red colors represent the genes with higher expression, and the green colors represent the genes with lower expression. Colored bars indicate the expression level. (**B**) Differential gene volcano plot. The volcano plot of differentially expressed genes in C, H and M. Red dots represent the up-regulated genes, and blue dots show down−regulated genes. Gray dots represent genes that did not show obvious changes amongdifferential samples.

In the process of differential transcript analysis, the FPKM (fragments per kilobase of transcript per million fragments mapped) of cells in the three groups was normalized, DESeq2 software was used to set FPKM ≥ 1 and Q value < 0.05, and 492, 369, and 361 differentially expressed genes were obtained in the three groups (H/N, (M+H)/N, (M+H)/H) of sheep granulosa cells, respectively. The following parameters were set for the differentially expressed genes: FPKM ≥ 1, H/N-FPKM/(M+H)/N-FPKM/(M+H)/H-FPKM > 1, *p* < 0.05, and 98, 48, 109 differently expressed mRNA were obtained. Among them, 13 mRNAs were significantly upregulated in the H/N group and downregulated in the (M+H)/H group; 4 mRNAs were significantly downregulated in the H/N group and upregulated in the (M+H)/H group (Table 2). The differentially expressed genes in the (M+H)/H group caused by melatonin mainly reflect changes in genes that protect against H_2_O_2_ damage (Figure 2).

### 3.3. Functional Enrichment and Signal Pathway Analysis

A GO functional enrichment analysis of 98, 48, and 109 differentially expressed genes obtained from the comparison of the H/N, (M+H)/N and (M+H)/H groups was performed using GOseq software, and three large categories were obtained. Among the 49 enriched functions in the H/N group, biological processes accounted for 46.9%, cell components accounted for 30.6%, and molecular functions accounted for 32.7%. Among the 47 enriched functions in the (M+H)/N group, biological processes accounted for 46.8%, cell components accounted for 46.8%, and molecular functions accounted for 23.4%. Among the 46 enriched functions in the (M+H)/H group, biological processes accounted for 50.0%, cell components accounted for 30.4%, and molecular functions accounted for 19.6%.

**Figure 3:**
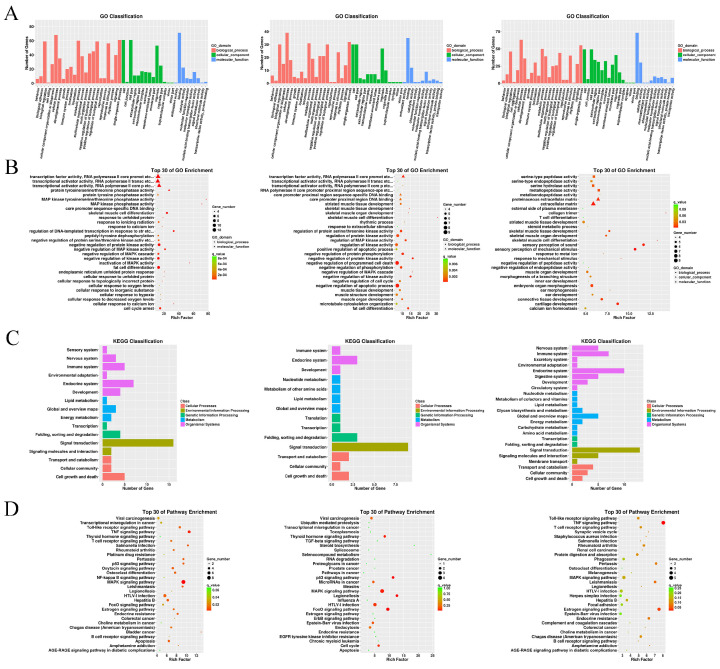
GO and KEGG analysis. GO-enriched bar graph of differentially expressed genes in granulosa cells with the control, H_2_O_2_ treatment and H_2_O_2_+Melatonin treatment. (**A**) The red bar indicates biological processes; green indicates cellular components; blue indicates molecular functions. (**B**) The scatter plot shows 30 GO terms in granulosa cells with the control, H_2_O_2_ treatment and H_2_O_2_+Melatonin treatment. (**C**) The ordinate indicates the GO terms, and the abscissa indicates the Rich factor. The size of the point indicates how many differentially expressed genes are in the GO terms, and the color of the point corresponds to the *p*.adj value range. The top 30 KEGG enrichment scatter plot indicates differentially expressed genes in granulosa cells with control, H_2_O_2_ treatment and H_2_O_2_+Melatonin treatment. (**D**) The ordinate indicates the KEGG terms, and the abscissa indicates the Rich factor. The size of the point indicates how many differentially expressed genes are in the KEGG pathways, and the color of the point corresponds to the *p*.adj value range.

A KEGG signaling pathway analysis was performed using KOBAS software on the differentially expressed genes obtained by comparing the H/N, (M+H)/N and (M+H)/H groups, and 24, 14 and 16 pathways were identified, respectively. Among them, the signal transduction pathway was the most enriched, with 18 in the (M+H)/H group. In addition, five genes were also found to be involved in the growth and development of granulosa cells (Figure 3).

### 3.4. RT–qPCR Validation

**Figure 4:**
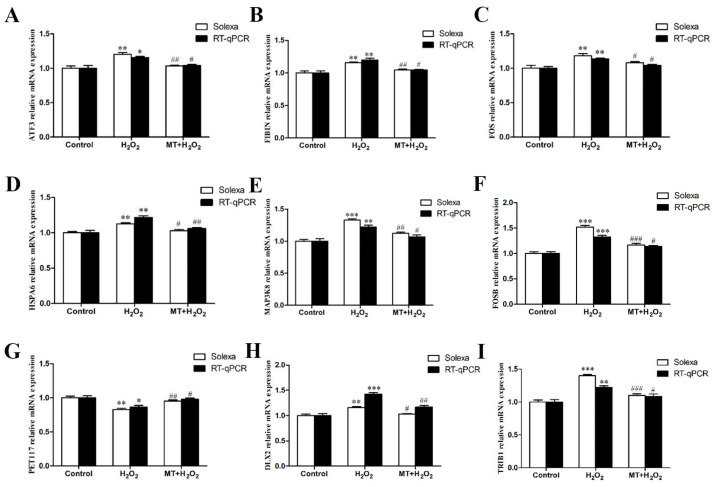
Comparison of the genes’ mRNA expression levels between high-throughput sequencing and qRT-PCR. (**A**–**I**): ATF3, FIBIN, FOS, HSPA6, MAP3K8, FOSB, PET117,DLX2 and TRIB1. The white bar represents high-throughput sequencing and the black bar represents qRT-PCR. The results were acquired from three independent experiments and are presented as the mean ± SD. For * *p* < 0.05 (***,** *p* < 0.01) vs. the control group, the difference is significant or extremely significant; for F # *p* < 0.05 (###,## *p* < 0.01) vs. the H_2_O_2_ group, the difference is significant or extremely significant.

Through functional analysis, we identified nine genes that may be closely related to the protective effect of melatonin against oxidative damage in sheep granulosa cells. Among them, ATF3, FIBIN, FOS, HSPA6, MAP3K8, FOSB, DLX2, and TRIB1 were upregulated in the H group and downregulated in the M+T group; PET117 was downregulated in the H group and upregulated in M+T group. The RT–qPCR results indicated that although the expression levels of genes in each group were significantly different, the expression trend was consistent with the high-throughput sequencing results (Figure 4).

### 3.5. Effect of Candidate Genes on Sheep Granulosa Cell Apoptosis

**Figure 5:**
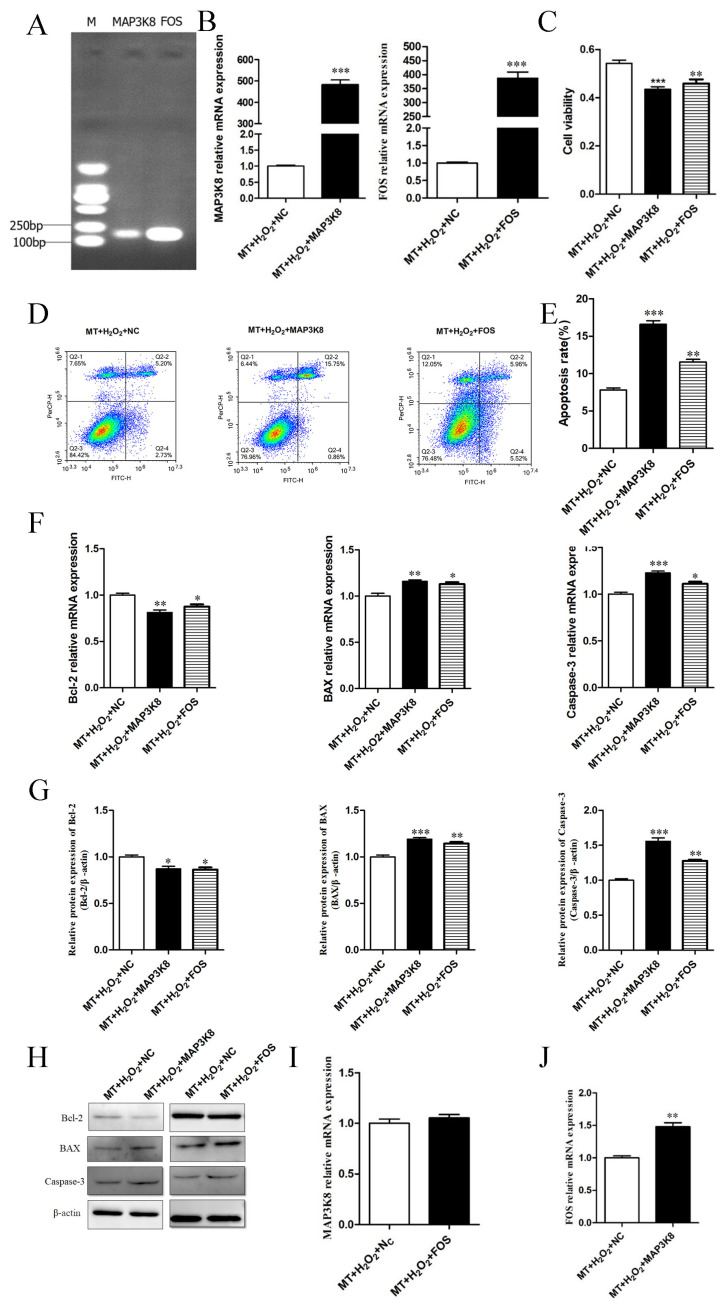
Melatonin inhibits H_2_O_2_-induced apoptosis of granulosa cells via MAP3K8 and FOS. (**A**,**B**) Construction of the overexpression vector of MAP3K8 and FOS. The expression of MAP3K8 and FOSB was determined by qRT-PCR; (**C**) Granulosa cells were transfected with MAP3K8 and FOS or negative control (NC). The viability of the different groups was measured by MTT; (**D**,**E**) Apoptosis levels measured by a flow cytometry assay. (**F**) The mRNA expression of Bax, Caspase-3 and Bcl-2 was determined by qRT-PCR analysis. (**G**,**H**) The protein expression of Bax, Caspase-3 and Bcl-2 was determined by western blot analysis. (**I**,**J**) Upstream and downstream relationship identification between MAP3K8 and FOS was measured by qRT-PCR. The results were acquired from three independent experiments and are presented as the mean ± SD. For * *p* < 0.05, the difference is significant; for ***,** *p* < 0.01, the difference is extremely significant.

Combined with KEGG analysis and the significance of gene expression differences, the MAPSK8 and FOS genes were screened as candidate genes for subsequent studies. After an overexpression vector of the candidate gene was successfully constructed, pcDNA3.1-MAP3K8 (referred to as MAP3K8), pcDNA3.1-FOS (referred to as FOS) and pcDNA3.1 (referred to as NC) were transfected into sheep granulosa cells, and 24 h later, cells were treated with H_2_O_2_ for 3 h, followed by the detection of apoptosis-related indicators. Compared with that in the control group, the viability of granulosa cells significantly decreased in both the MAP3K8 overexpression and FOS overexpression groups (*p* < 0.01), and the granulosa cell apoptosis rate significantly increased (*p* < 0.01). The expression levels of the proapoptotic genes Bax and Caspase-3 significantly decreased, and the expression level of Bcl2 significantly decreased (*p* < 0.05); the protein expression level was consistent with the mRNA level. Therefore, MAP3K8 and FOS gene overexpression can inhibit the viability of granulosa cells and promote granulosa cell apoptosis (Figure 5A–H).

As seen in Figure 5I–J, the increase in MAP3K8 expression significantly increased the expression of FOS (*p* < 0.01). Furthermore, the increase in FOS expression had no significant effect on the expression of MAP3K8 (*p* > 0.05), indicating that MAP3K8 was an upstream regulator of FOS expression.

## 4. Discussion

This study proves that melatonin inhibits the H_2_O_2_-induced apoptosis of sheep granulosa cells by suppressing oxidative stress via activation of the MAP3K8-FOS pathway. To the best of our knowledge, this is the first report revealing the inhibitory effect of melatonin on H_2_O_2_-induced apoptosis in sheep granulosa cells and the underlying mechanism of this effect.

Melatonin has been found in the follicles of many mammals, such as cattle [21] and swine [22]. It may participate in the protection of follicular cells from oxidative damage and play an important regulatory role in the maturation of oocytes. As a potent antioxidant, melatonin itself and its metabolites can protect the body from oxidative damage. Melatonin may act through PKA and PKB simultaneously in human granulosa cells to prevent follicular atresia and early luteinization at the antral stage [23]. Studies on the in vitro culture of porcine granulosa cells showed that, with the extension of the culture time, the endogenous melatonin content gradually decreased and the addition of melatonin to the culture medium effectively delayed the apoptosis of follicular granulosa cells and inhibited the apoptosis of granulosa cells and follicle atresia [8]. Melatonin attenuates palmitic acid-induced apoptosis in mouse granulosa cells through endoplasmic reticulum stress [24]. Melatonin attenuates deoxynivalenol-induced apoptosis in human granulosa cells [25] and affects the proliferation and apoptosis of sheep granulosa cells under heat stress [19]. The addition of melatonin and H_2_O_2_ affects the growth status of cells, and screening at different concentrations can optimize the effect. In the current study, the addition of 10 ng/mL melatonin effectively protected against sheep granulosa cell apoptosis caused by H_2_O_2_ (250 μM) damage. Therefore, melatonin can protect granulosa cells from apoptosis caused by a variety of exogenous stresses.

After the normal growth state of granulosa cells is stimulated by the external environment, the conformation of proapoptotic proteins changes, allowing them to transfer from the cytosol to the outer mitochondrial membrane and interact with the antiapoptotic proteins on and inside the membrane, eventually leading to apoptosis [26,27]. High-throughput sequencing identified 581 differentially expressed genes in melatonin-treated hen granulosa cells. These genes were mainly involved in biological processes such as cell proliferation and apoptosis. KEGG analysis revealed that these genes were mainly involved in the mTOR, PI3K-Akt, FoxO, and MAPK signaling pathways [28]. In the current study, 255 differentially expressed genes were selected from melatonin-treated and melatonin- and H_2_O_2_-treated granulosa cells by high-throughput sequencing; the expression levels of 17 genes changed significantly after melatonin treatment. This change indicates that melatonin plays a significant role in damage repair. The results of functional and KEGG analyses indicated that these differentially expressed genes were mainly involved in the MAPK, TNF, Toll-like receptor, and T-cell receptor-signaling pathways. We all know that a variety of genes are involved in cell damage and apoptosis in the MAPK and TNF pathways, and the addition of melatonin changes the gene expression in the above pathways, indicating that the regulatory pathway of melatonin in protecting the apoptosis function of granulosa cells is likely to be regulated by signals such as MAPK and TNF. In a previous study on the ameliorative effects of melatonin as an adjuvant for fertility preservation, it was shown that there was a significant downregulation in the mRNA expression of TNF-α, NF-Kβ, mTOR and p38-MAPK [29]. Therefore, melatonin is involved in many influencing factors and gene regulatory pathways in the regulation of granulosa cell apoptosis and can protect granulosa cells from apoptosis and alleviate the damage caused by exogenous stimuli through a variety of regulatory methods.

Melatonin can inhibit mitochondrial autophagy and inhibit FOXO1-mediated autophagy to protect mouse granulosa cells from oxidative damage [30]. Melatonin can enhance PDK1/Akt or PINK1/Parkin signaling in granulosa cells in polycystic ovary syndrome (PCOS) patients to alter the expression levels of the SIRT1 gene, thereby regulating mitochondrial function [31,32]. Melatonin can reduce hypoxia-induced apoptosis in granulosa cells by reducing ROS and activating the MTNR1B-PKA-Caspase8/9 pathway [33]. As important regulatory factors in the MAPK pathway, MAP3K8 and FOS are closely related to cell proliferation and apoptosis. MAP3K8 can inhibit monocyte apoptosis [34]. In myeloma tumor cells, MAP3K8 acts as a mitogen in mitosis, induces MAP3K, and inhibits tumor cell apoptosis [35]. Treatment with MAP3K8 inhibitors alone induced DNA fragmentation and poly (ADP-ribose) polymerase (PARP) cleavage of the two markers, indicating that apoptosis was induced in 8505C monolayer cells [36]. FOS alleviated apoptosis and rescued impaired GLP-1 release in TNF-α-treated L cells [37]. Furthermore, miR-29c-3p regulated FOS to repress EMT and cell proliferation and facilitate apoptosis [38]. In this study, MAP3K8 and FOS gene overexpression significantly increased the granulosa cell apoptosis rate, indicating that the two genes are upstream and downstream regulators. Therefore, it is proposed that melatonin can alleviate the apoptosis caused by H_2_O_2_ oxidative damage to granulosa cells by downregulating the expression of the MAP3K8 and FOS genes.

In summary, because MAP3K8 and FOS are important regulatory genes in the MAPK signaling pathway, we inferred that melatonin alleviates the proapoptotic injury induced by H_2_O_2_ in sheep granulosa cells through MAP3K8-FOS in the MAPK signaling pathway. The results of this study provide another theoretical basis for the mechanism of action of melatonin in alleviating oxidative stress in granulosa cells.

## Figures and Tables

**Table 1:** Primer information for quantitative real-time polymerase chain reaction.

Genes	Primer Sequence (5’–3’)	Size (bp)
FOSB	F: TCCGCCGAGTCTCAGTATCTGTC R: TGGAGGTCCTGGCTGGTTGTG	146
TRIB1	F: GACGGGAGCCTTCGGAGAGAC R: GTCACTGTCCTCCTGGTACTCTGG	140
DLX2	F: TTGACAGTCTGGTGGCTGATATGC R: GCTTGTGGAGGCTGCTGCTG	129
PET117	F: GAGACGCTCCAAGGTGGTGTTG R: GTCCTGCCGCTGCTTCAGATG	85
MAP3K8	F: ACAACGAGGAGGAGCGGTCTG R: ACGCTTCGCAGTGTTGGATATGT	131
HSPA6	F: AAGGAGACGGCGGAGGCTTAC R:GCTGCGAGTCGTTGAAGTAGGC	82
FOS	F: CACCGACCTGCCTGCAAGATC R: GCTCCATGCTGCCGACACTC	193
FIBIN	F:CAGGCTCAACGAGGACTTCTTGG R:CATGGCTCCTGATGGTGAGTGC	125
ATF3	F: GAAGTCAGTGCCTCTGCCATCG R: GGCGAACCTCAGCTCTTCCTTG	108
Bcl2	F: CGCATCGTGGCCTTCTTT R: CGGTTCAGGTACTCGGTCATC	113
Bax	F: CGAGTGGCGGCTGAAAT R: GGTCTGCCATGTGGGTGTC	237
β-actin	F: GCAAAGACCTCTACGCCAAC R: GGGCAGTGATCTCTTTCTGC	90

**Table 2:** 17 genes in the H/N comparisons that are regulated in the opposite way in the (M+H)/H comparisons.

Gene	Full Genetic Name	*p*-Value	Error Discovery Rate	Log2	Up/ Down
UNC13C	unc-13 homolog C	4.03 × 10^−16^	1.69 × 10^−12^	2.035441	UP
STAR	steroidogenic acute regulatory protein	1.13 × 10^−11^	1.88 × 10^−08^	1.465299	UP
MMP9	matrix metallopeptidase 9	6.34 × 10^−08^	4.42 × 10^−05^	1.785441	UP
PET117	collagen type II alpha 1 chain	6.12 × 10^−08^	4.42 × 10^−05^	4.656607	UP
HSPA6	heat shock protein family A (Hsp70) 6member 6	2.68 × 10^−29^	4.49 × 10^−25^	−2.76246	DOWN
FGL2	fibrinogen like 2	2.12 × 10^−25^	1.77 × 10^−21^	−2.2515	DOWN
TRIB1	tribbles pseudokinase 1	4.25 × 10^−17^	2.37 × 10^−13^	−1.75357	DOWN
FOS	Fos proto-oncogene, AP-1 transcription factor subunit	1.94 × 10^−15^	5.43 × 10^−12^	−1.57275	DOWN
FOSB	Fos proto-oncogene B, AP-1 transcription factor subunit	5.24 × 10^−13^	1.25 × 10^−09^	−2.60732	DOWN
MAK3P8	mitogen-activated protein kinase kinase kinase 8	1.32 × 10^−12^	2.77 × 10^−09^	−3.29209	DOWN
SFRP4	secreted frizzled related protein 4	5.21 × 10^−12^	9.70 × 10^−09^	−1.6262	DOWN
JUN	Jun proto-oncogene, AP-1 transcription factor subunit	4.70 × 10^−10^	7.15 × 10^−07^	−1.1781	DOWN
ATF3	Activating transcription factor 3	5.95 × 10^−08^	4.42 × 10^−05^	−1.347907	DOWN
FIBIN	fin bud initiation factor homolog (zebrafish)	2.54 × 10^−09^	3.28 × 10^−06^	−1.1054	DOWN
STC1	stanniocalcin 1	1.12 × 10^−07^	7.49 × 10^−05^	−1.283589	DOWN
TGM2	transglutaminase 2	1.43 × 10^−07^	9.19 × 10^−05^	−1.092309	DOWN
DLX2	distal-less homeobox 2	4.64 × 10^−09^	5.55 × 10^−06^	−2.56006	DOWN

## Data Availability

Not applicable.

## References

[1] Reiter R.J., Paredes S.D., Manchester L.C., Tan D.X. (2009). Reducing oxidative/nitrosative stress: A newlydiscovered genre for melatonin. Crit. Rev. Biochem. Mol. Biol..

[2] Reiter R.J., Tamura H., Tan D.X., Xu X.Y. (2014). Melatonin and the circadian system: Contributions to successful female reproduction. Fertil. Steril..

[3] Tamura H., Takasaki A., Taketani T., Tanabe M., Kizuka F., Lee L., Tamura I., Maekawa R., Asada H., Yamagata Y. (2013). Melatonin as a free radical scavenger in the ovarian follicle. Endocr. J..

[4] Hussein M.R., Bedaiwy M.A., Falcone T. (2006). Analysis of apoptotic cell death, Bcl-2, and p53 protein expression in freshly fixed and cryopreserved ovarian tissue after exposure to warm ischemia. Fertil. Steril..

[5] Barberino R.S., Menezes V.G., Ribeiro A.E., Palheta R.C., Jiang X., Smitz J.E., Matos M.H.T. (2017). Melatonin protects against cisplatin-induced ovarian damage in mice via the MT1 receptor and antioxidant activity. Biol. Reprod..

[6] Talpur H.S., Worku T., Rehman Z.U., Dad R., Bhattarai D., Bano I., Farmanullah, Liang A., He C., Yang L. (2017). Knockdown of melatonin receptor 1 and induction of follicle-stimulating hormoneon the regulation of mouse granulosa cell function. Reprod. Biol..

[7] Lin T., Lee J.E., Kang J.W., Oqani R.K., Cho E.S., Kim S.B., Jin D. (2018). Melatonin supplementation during prolonged in vitro maturation improves the quality and development of poor-quality porcine oocytes via anti-oxidative andanti-apoptotic effects. Mol. Reprod. Dev..

[8] He Y., Deng H., Jiang Z., Li Q., Shi M., Chen H., Han Z. (2016). Effects of melatonin on follicular atresia and granulosa cell apoptosis in the porcine. Mol. Reprod. Dev..

[9] Wang Y., Zeng S. (2018). Melatonin promotes ubiquitination of phosphorylated pro-apoptotic protein Bcl2-interacting mediator of cell death-extra long (BimEL) in porcine granulosa cells. Int. J. Mol. Sci..

[10] Fang Y., Deng S., Zhang J., Liu H., Li Y., Zhang X., Liu Y. (2018). Melatonin-mediated development of ovine cumulus cells, perhaps by regulation of DNA methylation. Molecules.

[11] Riaz H., Yousuf M.R., Liang A., Hua G.H., Yang L. (2019). Effect of melatonin on regulation of apoptosis and steroidogenesis in cultured buffalo granulosa cells. Anim. Sci. J..

[12] Zhao S.-Q., Zhang Y., Gao Y., Yang X.-P., Yang Z., Yang Z.-J. (2021). The in vitro effects of melatonin and Cry gene on the secretion of estradiol from camel ovarian granulosa cells. Domest. Anim. Endocrinol..

[13] Liu Y., Yang Y., Li W., Ao H., Zhang Y., Zhou R., Li K. (2019). Effects of melatonin on the synthesis of estradiol and gene expression in pig granulosa cells. J. Pineal Res..

[14] Perego M.C., Bellitto N., Maylem E.R.S., Caloni F., Spicer L.J. (2021). Effects of selected hormones and their combination on progesterone and estradiol production and proliferation of feline granulosa cells cultured in vitro. Theriogenology.

[15] Wang S.J., Liu W.J., Wu C.J., Ma F.H., Ahmad S., Liu B.R., Han L., Jiang X.P., Zhang S.J., Yang L.G. (2012). Melatonin suppresses apoptosis and stimulates progesterone production by bovine granulosa cells via its receptors (MT1 and MT2). Theriogenology.

[16] Hao E.-Y., Wang D.-H., Chang L.-Y., Huang C.-X., Chen H., Yue Q.-X., Zhou R.-Y., Huang R.-L. (2020). Melatonin regulates chicken granulosa cell proliferation and apoptosis by activating the mTOR signaling pathway via its receptors. Poult. Sci..

[17] Wu G., Song D., Wei Q., Xing J., Shi X., Shi F. (2018). Melatonin mitigates bisphenol A-induced estradiol production and proliferation by porcine ovarian granulosa cells in vitro. Anim. Reprod. Sci..

[18] Nakamura E., Otsuka F., Terasaka T., Inagaki K., Hosoya T., Tsukamoto-Yamauchi N., Toma K., Makino H. (2014). Melatonin counteracts BMP-6 regulation of steroidogenesis by rat granulosa cells. J. Steroid Biochem. Mol. Biol..

[19] Fu Y., He C.-J., Ji P.-Y., Zhuo Z.-Y., Tian X.-Z., Wang F., Tan D.-X., Liu G.-S. (2014). Effects of melatonin on the proliferation and apoptosis of sheep granulosa cells under thermal stress. Gene.

[20] Li X., Xu G., Li Z., Liu H., Ma X., Yang L., Zhang P., Zhao J., Wang J., Lu W. (2021). Gonadotropin-Inhibiting Hormone Promotes Apoptosis of Bovine Ovary Granulosa Cells. Life Sci..

[21] Tian X., Wang F., He C., Zhang L., Tan D., Reiter R.J., Xu J., Ji P., Liu G. (2014). Beneficial effects of melatonin on bovine oocytes maturation: A mechanistic approach. J. Pineal Res..

[22] Shi J.-M., Tian X.-Z., Zhou G.-B., Wang L., Gao C., Zhu S.-E., Zeng S.-M., Tian J.-H., Liu G.-S. (2010). Melatonin exists in porcine follicular fluid and improves in vitro maturation and parthenogenetic development of porcine oocytes. J. Pineal Res..

[23] Asma A., Marc-André S. (2022). Melatonin Signaling Pathways Implicated in Metabolic Processes in Human Granulosa Cells (KGN). Int. J. Mol. Sci..

[24] Chen Z., Lei L., Wen D., Yang L. (2019). Melatonin attenuates palmitic acid-induced mouse granulosa cells apoptosis via endoplasmic reticulum stress. J. Ovarian Res..

[25] Xue R., Li S., Zou H., Ji D., Lv M., Zhou P., Wei Z., Zhang Z., Cao Y. (2021). Melatonin alleviates deoxynivalenol-induced apoptosis of human granulosa cells by reducing mutually accentuated FOXO1 and ER stress. Biol. Reprod..

[26] Basini G., Simona B., Santini S.E., Grasselli F. (2008). Reactive oxygen species and anti-oxidant defences in swine follicular fluids. Reprod. Fertil. Dev..

[27] Agarwal A., Gupta S., Sharma R.K. (2005). Role of oxidative stress in female reproduction. Reprod. Biol. Endocrinol..

[28] Hao E. (2020). Research on the Regulatory Mechanism of mTOR Signaling Pathway Mediated Melatonin to Delay Ovary Aginginlate-Phase Laying Hen. Ph.D. Thesis.

[29] Al-Shahat A., Hulail M.A.E., Soliman N.M.M., Khamis T., Fericean L.M., Arisha A.H., Moawad R.S. (2022). Melatonin Mitigates Cisplatin-Induced Ovarian Dysfunction via Altering Steroidogenesis, Inflammation, Apoptosis, Oxidative Stress, and PTEN/PI3K/Akt/mTOR/AMPK Signaling Pathway in Female Rats. Pharmaceutics.

[30] Shen M., Cao Y., Jiang Y., Wei Y., Liu H. (2018). Melatonin protects mouse granulosa cells against oxidative damage by inhibiting FOXO1-mediated autophagy: Implication of an antioxidation-independent mechanism. Redox Biol..

[31] Zheng B., Meng J., Zhu Y., Ding M., Zhang Y., Zhou J. (2021). Melatonin enhances SIRT1 to ameliorate mitochondrial membrane damage by activating PDK1/Akt in granulosa cells of PCOS. J. Ovarian Res..

[32] Yi S., Zheng B., Zhu Y., Cai Y., Sun H., Zhou J. (2020). Melatonin ameliorates excessive PINK1/Parkin-mediated mitophagy by enhancing SIRT1 expression in granulosa cells of PCOS. Am. J. Physiol. Endocrinol. Metab..

[33] Tao J.-L., Zhang X., Zhou J.-Q., Li C.-Y., Yang M.-H., Liu Z.-J., Zhang L.-L., Deng S.-L., Zhang L., Shen M. (2021). Melatonin Alleviates Hypoxia-Induced Apoptosis of Granulosa Cells by Reducing ROS and Activating MTNR1B-PKA-Caspase8/9 Pathway. Antioxidants.

[34] Sanz-Garcia C., Sánchez Á., Contreras-Jurado C., Cales C., Barranquero C., Muñoz M., Merino R., Escudero P., Sanz M.-J., Osada J. (2017). MAP3K8 Modulates Monocyte State and Atherogenesis in ApoE-/- Mice. Arter. Thromb. Vasc. Biol..

[35] Hebron E., Hope C., Kim J., Jensen J.L., Flanagan C., Bhatia N., Maroulakou I., Mitsiades C., Miyamoto S., Callander N. (2013). MAP3K8 kinase regulates myeloma growth by cell-autonomous and non-autonomous mechanisms involving myeloma-associated monocytes/macrophages. Br. J. Haematol..

[36] Gianì F., Russo G., Pennisi M., Sciacca L., Frasca F., Pappalardo F. (2019). Computational modeling reveals MAP3K8 as mediator of resistance to vemurafenib in thyroid cancer stem cells. Bioinformatics.

[37] Wongkrasant P., Pongkorpsakol P., Chitwattananont S., Satianrapapong W., Tuangkijkul N., Muanprasat C. (2020). 4 Fructo-oligosaccharides alleviate inflammation- associated apoptosis of GLP-1 secreting L cells via inhibition of iNOS and cleaved caspase-3 expression. J. Pharm. Sci..

[38] Yao L., Yang L., Song H., Liu T., Yan H. (2020). MicroRNA miR-29c-3p modulates FOS expression to repress EMT and cell proliferation while induces apoptosis in TGF-β2-treated lens epithelial cells regulated by lncRNA KCNQ1OT1. Biomed. Pharmacother..

